# The effect of water immersion on vection in virtual reality

**DOI:** 10.1038/s41598-020-80100-y

**Published:** 2021-01-13

**Authors:** Géraldine Fauville, Anna C. M. Queiroz, Erika S. Woolsey, Jonathan W. Kelly, Jeremy N. Bailenson

**Affiliations:** 1 Department of Communication, Stanford University, Stanford, USA; 2 The Hydrous, Sausalito, USA; 3 Department of Psychology, Iowa State University, Ames, USA

**Keywords:** Psychology, Human behaviour

## Abstract

Research about vection (illusory self-motion) has investigated a wide range of sensory cues and employed various methods and equipment, including use of virtual reality (VR). However, there is currently no research in the field of vection on the impact of floating in water while experiencing VR. Aquatic immersion presents a new and interesting method to potentially enhance vection by reducing conflicting sensory information that is usually experienced when standing or sitting on a stable surface. This study compares vection, visually induced motion sickness, and presence among participants experiencing VR while standing on the ground or floating in water. Results show that vection was significantly enhanced for the participants in the Water condition, whose judgments of self-displacement were larger than those of participants in the Ground condition. No differences in visually induced motion sickness or presence were found between conditions. We discuss the implication of this new type of VR experience for the fields of VR and vection while also discussing future research questions that emerge from our findings.

## Introduction

Multiple sensory modalities such as vision, audition, the inner ear vestibular system, the skin’s somatosensory system and the proprioceptive system of muscles and joints constantly helps us make sense of our movements in the world^1^. The conscious and subjective experience of self-motion, called vection, is induced by the integration of cues from these sensory modalities^2^. This project adds to the literature on multisensory contributions to vection by comparing vection experienced through virtual reality when floating in water and when standing on the ground.

### Sensory inputs to vection

Although multiple sensory modalities are involved in vection, vision plays a predominant role^3,4^. A popular example of vection induced by visual input is the train illusion which occurs when a person sitting on a stationary train observes the neighboring train leaving the station, inducing erroneous vection. The study of vection started almost 150 years ago with Mach’s study^5^ in which he placed stationary seated participants in a large drum rotating around a vertical axis with patterns on the inside, triggering an illusion of self-rotation. Since then scholars have investigated the influence of different aspects of optical flow on vection, such as speed^6,7^, pattern size and density^7,8^, location and size of the retinal areas stimulated^6^, and addition of jitter and oscillation to the visual field^9–11^. Besides the importance of optic flow in inducing vection, researchers have also highlighted the role played by perceived surface properties^12,13^. For example, researchers invited participants to watch a display presenting motion through 3D tunnels constructed from various materials. The results indicated that bark, fabric, stone, wood and leather induced stronger vection than ceramic, glass, fur and metal. They argued that the surface properties of the materials modulate vection as they showed that perceived depth, smoothness and rigidity were related to vection strength^12^. Non-visual contributions to vection have also been investigated in a wide range of settings. To explore the role of the vestibular system, participants have adopted different positions such as standing, sitting or supine along with different body tilt^14–16^ and have flown on parabolic flights, which simulate zero gravity^17–19^. Researchers have also looked at the role that the vestibular system could play in decreasing the latency of vection. In the natural environment body movements directly trigger perceived self-motion^20^ while between one to ten seconds are needed to trigger vection after the onset of visual motion^21^. This latency seems associated with the sensory conflict between the visual and non-visual cues with the vestibular information being crucial^22^. Vibration and Vestibular stimulation have been shown to reduce vection latency as argued that “adding noise to the vestibular system reduces the reliance on vestibular cues for self-motion perception”^21, p. 82^.

Scholars have exposed different surfaces of participants’ skin to wind in order to understand the role played by the somatosensory system^23,24^. The proprioceptive system has been primarily studied in relation to walking movements^23,25,26^. Attention has also been given to arm movements (e.g., standing and making breaststroke motions^27^;). Finally, auditory stimuli, often in the form of virtual spatialized sound presented through headphones, produces relatively weak vection on its own but contributes significantly to vection when presented with visual motion^28–30^.

Many measures of vection have been applied across the field, such as the number of instances of reported vection per stimulus, vection onset time^7,31^, vection intensity^7,32^ and convincingness of vection^33^. Besides self-report measures of vection per se, other researchers have used measures that emphasize the downstream effects of vection, such as perceived displacement^32,34^, perceived body tilt, or perceived velocity^35^. Moreover, visual motion induces actual head and body displacement that can be measured^3,36^.

In order to present the visual input to their participants, studies on vection have used various displays such as rotating cylinders with the participants seated in the center and looking at the cylinder rotating horizontally around them^8^, flat screens^37^, or curved screens^29^. Recently, virtual reality (VR) head-mounted displays have been used to study vection^32^.

Previous scholars (^38, p. 47^) reminded us that vection “can be affected by a wide range of parameters including attention, viewing patterns, the perceived depth structure of the stimulus, perceived foreground/background distinction (even if there is no physical separation), cognitive-perceptual frameworks, ecological validity, as well as spatial presence and involvement.” They encouraged more basic and applied research in order to “come closer to fulfilling the promise of VR as an alternate reality, that enables us to perceive, behave, and more specifically locomote and orient as easily and effectively in virtual worlds as we do in our real environment” (ibid). In this way, it would be interesting to explore how floating in water could transform vection while in VR compared to standing on the ground. Participants floating in water might experience greater vection as the proprioceptive inputs are ambiguous when floating, whereas in standing position the proprioceptive system (of muscles, joints and tendons) would be providing biomechanical information about the stationary position.

### Relationship between vection, VIMS, and presence

Visually induced motion sickness (VIMS) is an overarching term for motion sickness symptoms driven by visual simulation in the absence of physical motion. Cyber sickness or simulator sickness are subcategories of VIMS dependent on the equipment used, in this case respectively VR and flight simulators. According to the sensory conflict theory, VIMS would be mainly caused by a conflict between the sensory inputs from the visual, vestibular, proprioceptive, and somatosensory systems^39,40^. This theory indicates a relationship between vection and VIMS, whereby reducing sensory conflict should facilitate vection and reduce VIMS^41^. As the vestibular system seems to play a key role in vection and in its latency due to sensory conflict between the visual and the vestibular system, it has been argued that the vestibular system might be important in the occurrence of VIMS. Researchers observed lower simulator sickness scores for participants receiving vestibular stimulation coupled with the visual motion, in this way suggesting that noisy vestibular stimulation can reduce simulator sickness^42^.

Other researchers have also highlighted a complex and weak relationship between vection and VIMS^43^. The Postural Instability Theory also describes the relationship between vection and VIMS, but emphasizes the motor control system rather than sensory conflict^44^. This theory suggests that changes in one’s postural stability is linked to VIMS, such that sickness is often preceded by increased head and body sway. According to this theory, people with weak postural control are at greater risk of suffering from VIMS. Along the same lines, environments that lead to poor postural control will trigger VIMS more easily. Vection triggers situations in which maintaining postural control becomes increasingly difficult, thus potentially triggering VIMS.

While many VR experiences aim to produce a compelling sense of vection among the users, it comes with the risk of inducing VIMS. In order to reduce the likelihood of VIMS, VR researchers and developers have shown a great deal of imagination to decrease conflict between the different sensory systems. To do so, they have investigated ways for the users to be physically active during exploration of a large virtual environment while preventing large displacement in the physical world. These strategies included walking in place^45,46^, stepping in place in a human-size hamster ball^47^, or walking on a directional treadmill^48,49^ or multidirectional surface made of tiles moving in the opposite direction of the user^50^. Another strategy, called redirected walking, consists of curving the walking trajectory through visual rotation of the virtual environment without being noticed by the users^51,52^ allowing them to explore a large virtual area while walking only in a limited space.

The feeling of being immersed and spatially present in an environment, a phenomenon called presence^53^, has been proposed by Riecke and colleagues^54^ to be influenced by vection as they demonstrated an increased spatial presence correlated with an enhanced convincingness of vection. Scholars^55^ supported this connection by demonstrating an association between vection and presence as well. While some researchers^54,55^ focused on visually-induced vection, others^56^ demonstrated a similar connection between presence and auditorily-induced vection.

The current body of research on the association between presence and VIMS tends to indicate a negative relationship between presence and cybersickness^57^. The mechanisms responsible for this inverse association could be attributed to the sensory conflict theory since when experiencing great presence, the participant’s attention might be directed away from the sensory conflict^58^. Moreover, this negative relationship seems to be mediated by vection, among other factors.

Latency, “the delay between a user’s action/motion and when that action is visible in the display”^59, p. 142^, is yet another important factor to consider in the relationship between vection, VIMS and presence as increased latency can decrease presence^60^. Moreover, Kim et al.^61^ exposed participants in VR to various levels of latency in pitch in order to study the effect of sensory conflict on presence, cybersickness and spatial perception. Their results indicated that cybersickness would increase while presence would decrease as a result of increased latency. The existing body of research indicates a complex relationship between vection, VIMS and presence, leading Weech and colleagues^57^ to encourage scholars to measure these three variables to further understand their relationship.

## Research goals

Research into understanding vection, with and without VR, has taken various forms, employed expensive equipment and sophisticated methods (such as parabolic flights), and experimented with a wide range of sensory cues. This study utilizes waterproof VR head mounted displays to understand the effects of aquatic immersion on vection, VIMS and presence.

The study has three objectives. The first objective is to present and demonstrate feasibility of a new kind of VR experience, and share a detailed research method. Despite the numerous ingenious strategies designed to better align the physical activity of the participants to the visual motion experiences in VR while immersed in water have never been tested before, to the best of our knowledge. This paper aims to demonstrate the feasibility of studying the impact of VR with participants immersed in water and offers a recipe to help future researchers design aquatic VR studies.

The second objective is to present to the vection research community a new strategy to test vection while keeping the participants stationary and minimizing contact with the stable environment. Vection researchers have been forward thinkers with advanced methods in order to dissect the role of different sensory systems on vection. We aim to demonstrate that water can be the new frontier for vection research, as it will offer new possibilities to test the role played by the different sensory systems in ways that are difficult on the ground. In particular, floating in water removes contact between the viewer and the stable real environment, which would otherwise conflict with visually presented self-motion.

The third objective is to contribute to the vection literature by providing the first empirical results comparing vection when immersed in water and when standing on the ground. These first results offer a unique opportunity to raise new research questions and reflect on the potential ways forward for this new field of research.

## Results

### Vection

**Figure 1:**
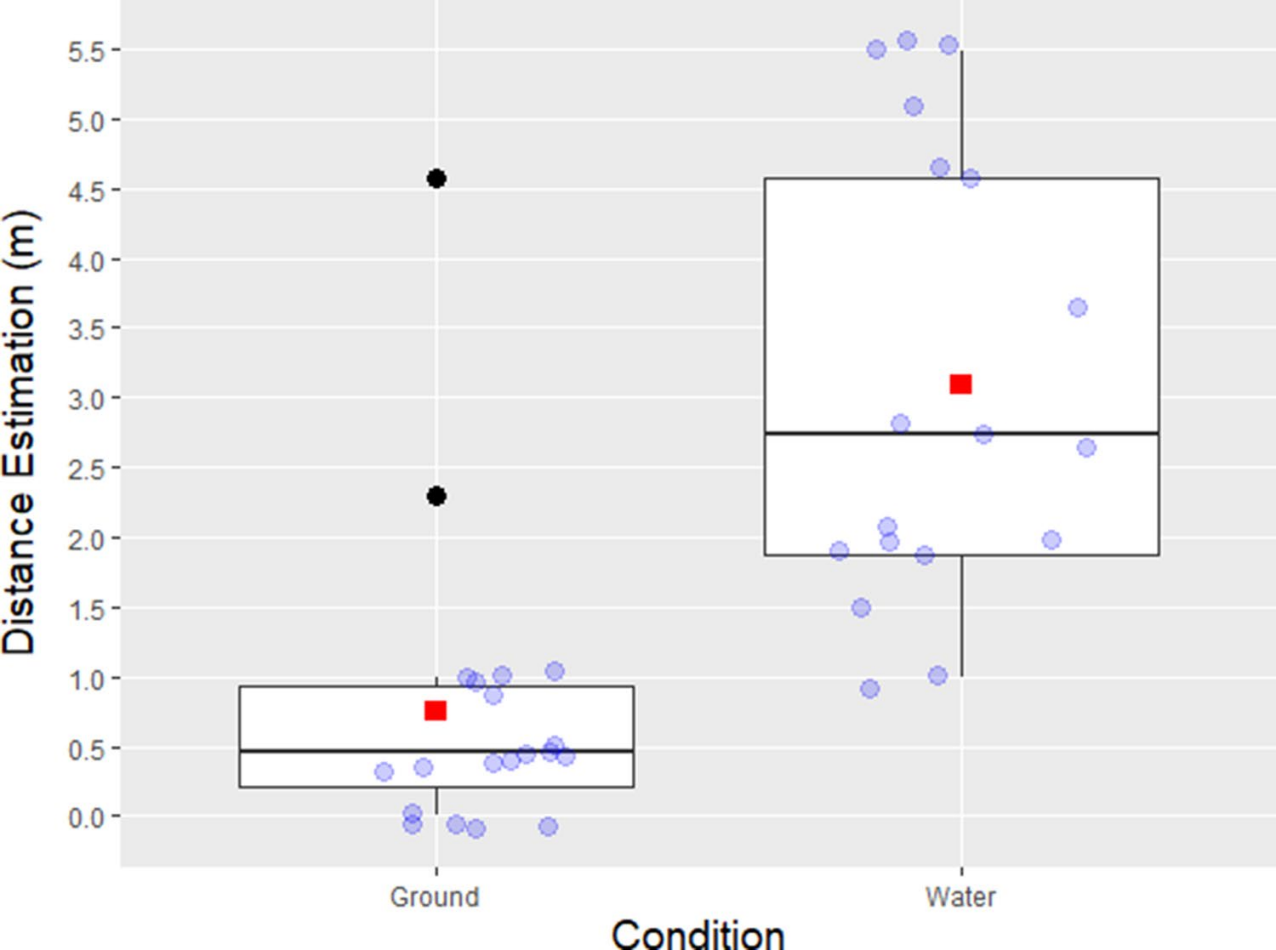
Comparison of the distance estimation in meters between Ground and Water conditions. Boxplot presenting medians, interquartile ranges, minimum and maximum and potential outliers. The boxplot identified the means (red squares) for each condition and two outliers (black dots) in the Ground condition.

All analyses were carried out in R version 3.5.2. A Shapiro–Wilk Normality test^62^ indicated significant deviations from normal distribution (*p* < 0.001), which can be observed in the data distribution shown in Fig. 1. Consequently, a non-parametric Kruskal–Wallis one-way analysis of variance was conducted. The analysis revealed a significant difference of egocentric distance estimations (χ^2^ (14, *n* = 38) = 29.71, *p* = 0.008 n = 38) between Water (*M* = 3.10, *SD* = 1.61, *Mdn* = 2.74, *IQR* = 2.70) and Ground (*M* = 0.75, *SD* = 1.05, *Mdn* = 0.46, *IQR* = 0.73) conditions. Figure 1 presents the boxplot of the egocentric distance estimation per condition that reveals two outliers. Another Kruskal–Wallis test was run after removing the outliers and this test was also significant (χ^2^(13, *n* = 36) = 30.33, *p* = 0.004).

### Visually induced motion sickness

**Figure 2:**
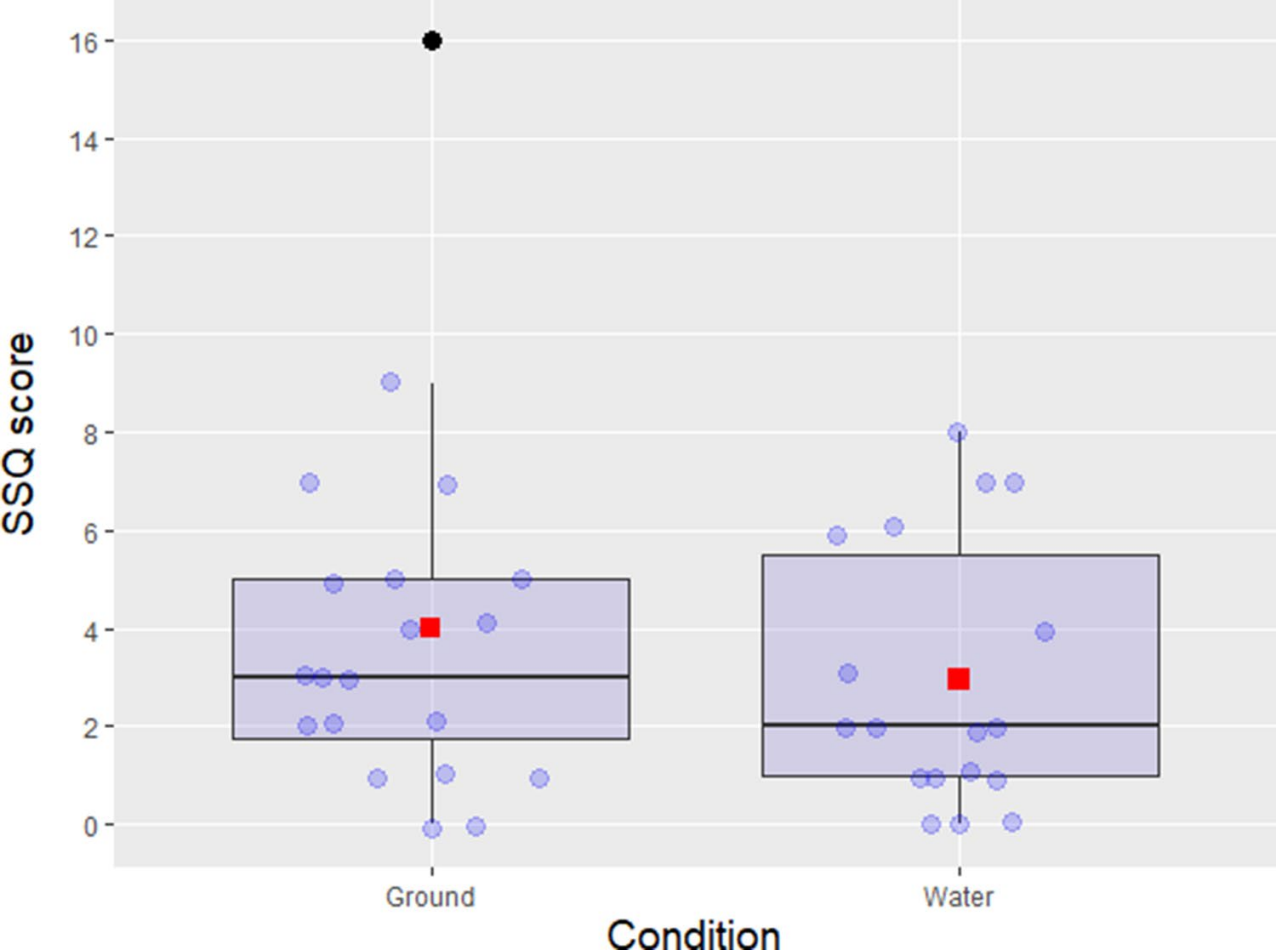
Comparison of the SSQ scores (sum of the 16 SSQ items) between Ground and Water conditions. Boxplot presenting medians, interquartile ranges, minimum and maximum and potential outliers. The boxplot also shows the means (red squares) for each condition and an outlier (black dot) in the Ground condition.

The scale reliability was good (Cronbach’s alpha = 0.73) and allowed to create an average score based on the sum of the 16 items from the SSQ (SSQ score). A Shapiro–Wilk Normality test^62^ indicated significant deviations from normal distribution (*p* < 0.001), which can be observed in Fig. 2 presenting the distribution of the data points. Consequently, a non-parametric Kruskal–Wallis test was conducted and no significant difference was found for the SSQ score (χ^2^ (1, *n* = 38) = 0.863, *p* = 0.353) between Water (*M* = 2.94, *SD* = 2.69, *Mdn* = 2, *IQR* = 4.50) and Ground (*M* = 4, *SD* = 3.73, *Mdn* = 3, *IQR* = 3.25) conditions (Fig. 2).

Exploratory analyses were conducted on individual questionnaire items. Setting alpha to 0.05 for each comparison, “eye strain” and “stomach awareness” were significantly greater for the Ground condition while “difficulty to concentrate” and “increased salivation” were significantly greater for the Water condition (Table 1). However, those differences are not significant after applying Bonferroni correction. A second exploratory approach was adopted by computing the Bayes factors for each SSQ item. Bayes factors associated with “eye strain” and “stomach awareness” items stand out as especially unlikely to be from the same distribution. This supports the significant difference that the Kruskal–Wallis test highlighted.

**Table 1:** Mean, standard deviation, median, interquartile range, Kruskal–Wallis’ χ^2^ (1, *n* = 38) and *p*-value, and Bayes factors for the 16 SSQ items for each condition.

Symptoms	Ground				Water				Kruskal–Wallis		Bayes factor
	Mean	SD	Mdn	IQR	Mean	SD	Mdn	IQR	*χ*^2^	*p*	BF
General discomfort	.5	.6	0	1	.44	.7	0	1	.26	.61	0.32
Fatigue	.2	.52	0	0	.05	.2	0	0	.92	.34	0.49
Headache	.15	.36	0	0	0	0	0	0	2.85	.09	1
Eyestrain	.6	.6	1	1	.17	.38	0	0	5.99	.01	4.2
Difficulty focusing	.4	.59	0	1	.38	.6	0	1	.01	.93	0.32
Increased salivation	.15	.36	0	0	.55	.7	0	1	.27	.04	2.19
Sweating	.05	.22	0	0	0	0	0	0	.9	.34	0.45
Nausea	.35	.58	0	1	.16	.38	0	0	1.02	.31	0.52
Difficulty concentrating	.1	.31	0	0	.39	.5	0	1	4.26	.04	1.88
Fullness of head	.2	.41	0	0	.11	.32	0	0	.55	.46	0.39
Blurred vision	.25	.44	0	0.25	.33	.68	0	0	0	1	0.34
Dizzy (eyes open)	.35	.67	0	0.25	.16	.38	0	0	.55	.46	0.47
Dizzy (eyes closed)	.1	.45	0	0	.11	.32	0	0	.39	.53	3.32
Vertigo	.25	.55	0	0	.05	.24	0	0	1.74	.19	0.67
Stomach awareness	.35	.49	0	1	0	0	0	0	7.52	.01	9.27
Burping	0	0	0	0	0	0	0	0	NaN	NA	NA

### Relationship between presence, VIMS and vection

Non-parametric Spearman’s rank correlation analyses were performed on the variables in order to evaluate their relationship in both conditions. As presented in Table 2, while most the relationships between the variables are weak and non-significant, it is interesting to notice the moderate but non-significant negative correlation between VIMS and presence.

**Table 2:** Correlation coefficients (rho) and associated *p* values for comparisons between VIMS, presence and vection.

Variables	Ground condition		Water condition	
	*rho*	*p*	*rho*	*p*
VIMS—Presence	− 0.42	0.06	− 0.04	0.87
Vection—VIMS	0.13	0.56	0.22	0.38
Vection—Presence	− 0.23	0.34	− 0.13	0.60

### Presence

A Shapiro–Wilk Normality test confirmed normal distribution of the presence scores (*p* = 0.592). There was no significant difference in presence (*t* = 0.986, *p* = 0.331, *n* = 38) between the Ground (*M* = 3.29, *SD* = 0.75) and the Water conditions (*M* = 3.5, *SD* = 0.55).

## Discussion

The contribution of this study is three-fold. First, this paper provides the first empirical results comparing vection, VIMS and presence felt by participants in VR either standing on the ground or floating in water. Second, this paper presents a new method to explore vection and offers new research questions in relation to water immersion. Third, as the field of VR is quickly expanding, this study presents and demonstrates the feasibility of a new kind of VR experience in water.

### Vection

In this study, we compared the impact of a VR experience on vection, VIMS and presence as the participants were either standing on the ground or floating in water. The results indicated enhanced vection in water as the distance estimation was significantly greater in the Water condition. Previous research on vection has described the relative contributions of different sensory modalities. In the case of this first study on vection while floating in water, it is important to discuss the different sensory inputs experienced by the participants in the two conditions.

The neutral buoyancy experienced when immersed in water has been widely used to study the physiological and psychological human reaction to weightlessness mimicking a reduced gravity field^63^. In this study, participants were placed in conditions mimicking different levels of gravity, from Earth gravity in the Ground condition to reduced gravity in the Water condition. The role of the visual-vestibular interaction has been studied in microgravity induced during parabolic flights when the vestibular system does not provide the “down” reference anymore^18,19^. In this case, the body relies more heavily on the visual input to evaluate self-motion, even during very brief exposure to microgravity^18^. Since weightlessness prevents appropriate vestibular input, other sensory systems such as vision are more heavily relied on^19^. Researchers^18^ demonstrated that vection magnitude as induced by visual stimuli is significantly greater during microgravity. Beside the greater reliance on the visual system when the vestibular system input is diminished during microgravity, Researchers^18^ also suggest that microgravity decreases the conflict between the visual and vestibular information. In this way, our findings align with previous studies on the positive impact of microgravity on vection, as the increased reliance on vision that occurs during parabolic flight also seems to occur during water immersion.

To the extent of our knowledge, the prone position adopted by participants in the Water condition has not been implemented in previous studies, making it difficult to infer the underlying mechanisms explaining how the different positions between Ground (standing) and Water (prone) conditions impacted vection. Although the prone position has not been previously tested, related research indicates that participants in supine position (lying on the back) demonstrate faster vection onset than seated participants, particularly for visual motion parallel to the ground^14^. Other research has shown that tilting the body away from vertical increases perceived vection parallel to the body axis^16^.

Second, the somatosensory input was different for our participants in the different conditions. The role of the somatosensory system has been explored by scholars^23^ investigating the impact of directional wind on the participants’ face suggesting forward self-motion combined with visual input. They found that participants experiencing visual and somatosensory motion cues experienced greater vection than participants experiencing visual cues only. Murata and colleagues^24^ combined somatosensory and proprioceptive systems while blindfolding the participants to avoid visual cues. The participants were seated on a horse-riding machine and swayed back and forth while directional wind was blowing on their whole body. Consistent with Seno’s and colleagues^23^ findings, the participants in the wind condition experienced greater vection than in the condition lacking somatosensory cues of motion. In our study, the participants in the Water condition had the somatosensory input from the water moving around their body. In this way, the water could have played a similar role than the wind previously used in studies^23,24^ and induced greater vection than in the Ground condition. Although in our case, it needs to be highlighted that the water movement was not directional.

Third, the proprioceptive system of muscles, joints, and tendons that provides biomechanical information about motion also contributes to vection and has been investigated mainly in relation to walking but also in relation to breaststroke movements. Researchers^28^ investigated how breaststroke movements with the arms and hands of standing participants would influence vection compared to passive viewing of an optic flow. They also investigated the impact of the congruence between the breaststroke movements and the optic flow. They showed that breaststroke movements (despite its congruence) increase vection compared to the passive condition and suggested that congruent breaststroke movement results in greater vection than incongruent movement. In our case, the participants in the Water condition were able to engage in swimming movements that could have a positive impact on vection. Participants’ behavior in the current study ranged from floating motionless to actively engaging in swimming motion, although these behaviors were not explicitly coded and analyzed. Another important proprioceptive difference between conditions is that participants in the Water condition had no contact with stable features of the environment. In contrast, participants in the Ground condition had constant contact between their feet and the stable ground, thereby providing biomechanical information about their stationary position.

The input from the different sensory systems involved in this study constitute a range of factors that could explain the significant difference in vection experienced between the two conditions. As this is the first study in the field of vection while floating in water while also in VR, this result opens up a wide range of new research questions that are now possible to investigate in an aquatic environment.

### Visually induced motion sickness (VIMS)

VIMS was measured with the commonly used Simulator Sickness Questionnaire (SSQ^64^) containing 16 items which can be separated in three subscales indicative for disorientation, oculomotor and nausea. As the reliability of two of the three subscales were too low, we looked primarily at the overall SSQ score where there was no significant difference between conditions. As this is the first time this scale is used in a floating situation, we decided to explore the 16 items individually. We found out that two items scored higher in the Water condition (“increased salivation” and “difficulty concentrating”) and two other scored higher in the Ground condition (“eyestrain” and “stomach awareness”), although these differences did not withstand correction for multiple comparisons.

The higher score for the “increased salivation” item for participants in Water condition could potentially be attributed to the different buccal situations in the two conditions. In the Water condition, participants wore a mouth piece and breathed through their mouth. The unfamiliarity of the equipment might contribute to less swallowing of the saliva compared to a more natural situation in the Ground condition. In this way, this item might not contribute to VIMS when the participants are equipped with a mouthpiece.

The higher score for the “difficulty concentrating” item for participants in Water condition could be attributed to the unusual situation in which the participants are finding themselves. As participants are floating in the pool with unfamiliar equipment, the “difficulty in concentrating” item might be an indication of something else than VIMS and might thus not be a reliable item in this specific context.

The higher score for the “eyestrain” item in participants in the Ground condition might be due to the fact that the headset, although using the same phone, was different thus potentially creating a discrepancy in ocular comfort between the conditions. As in the Water condition, the water itself serves as a lens that is not present in the Ground condition. This difference of equipment between conditions could explain the difference observed on this SSQ item.

The difference in “stomach awareness” scores between Ground and Water conditions could potentially be an indication of greater VIMS in the Ground condition. Standing on the ground while experiencing visual motion might trigger sensory conflict that has been argued to cause VIMS^39,40^. In this way, this could be the very first indication that experiencing VR while immersed in water might lessen VIMS. As described earlier, latency can also contribute to VIMS^22^. In this study, as the same device was used in both conditions, all the participants were exposed to the same potential effect of latency. Moreover, the latency of the Samsung S8 remains approximately below 20 ms, a level of latency that seems unlikely to cause VIMS, although latency perception varies among people^59,65^.

### Presence

Presence, the psychological state of feeling present in a virtual environment^66^ was measured with 6 questions. As previously demonstrated^67^ presence tends to be positively correlated to virtual immersion. As an example, in studies where participants are experiencing similar virtual activities with different levels of immersion such as VR headset versus a laptop computer, participants in the more immersive conditions tend to experience greater presence^68^. It has also been argued that a system is more likely to be immersive if it offers high fidelity simulation through multiple sensory modalities^66^. In this study, the participants in the Water condition were indeed subjected to a more immersive VR experience as the water immersion increased alignment between visual and non-visual sensory modalities. Surprisingly, the presence reported by the participants did not differ between conditions. Hypothetically, one can imagine that the participants in the Water condition might have been distracted by the equipment or that the lack of spatial grounding might have also had an impact on the presence. It would be interesting to investigate if giving them more time to acclimate to the procedure would mitigate this potential distraction. Further studies exploring the feeling of presence in aquatic VR are required to confirm our results.

### Relationship between vection, VIMS and presence

While researchers are still trying to clarify the relationship between vection, VIMS and presence, the sensory conflict theory seems to play a key role in making sense of these relationships. A negative relationship seems to exist between VIMS and vection^41^ and between VIMS and presence^57^ which seems to be mediated by vection. This study aimed at contributing to the knowledge concerning these relationships by exploring potential correlations between the three variables in the two conditions. While the results did not reveal any significant correlation, and thus prevent us from drawing any strong conclusion, VIMS and presence showed a moderate negative correlation consistent with past research^57^.

### Limitations and future studies

This very first study on the impact of aquatic VR on vection, VIMS and presence has some limitations that need to be addressed and taken into account in future studies. First, the study was canceled prematurely due to the COVID-19 pandemic. This resulted in a lower number of data points than we had hoped to collect, although the final data set is still larger than many studies published on the psychology of VR. For example, in Table 1 of the meta-analysis on immersion and presence by Cummings and Bailenson^67^, almost half (38 out of 83) studies had samples of 38 participants or fewer. Nonetheless, we recognize that we might be missing effects due to the low number of data points (type II error) and look forward to replicating and extending this work once the pandemic ends.

Another limitation might reside in the measurement tool used in this new environment to measure VIMS. The SSQ might not be the most appropriate tool for investigating VIMS in an aquatic environment and thus, we encourage future researchers to investigate other VIMS scales. A shorter scale focusing on the nausea aspect such as the misery scale^69^, adapted to study VIMS and cyber sickness^70,71^ might be a better choice as it focuses mainly on the stomach awareness aspect of motion sickness and should be investigated in future studies. The fast motion sickness scale, where the participants verbally rate the potential sickness from 0 (no sickness at all) to 20 (frank sickness) could also be used^72^. Besides being potentially more adapted to fit the reality of an experiment in the water, the misery scale and the fast motion sickness scale have the advantages of being shorter than the SSQ.

In this study, vection was measured solely through distance estimation. For future studies, an important addition to strengthen the measure of vection would be the intensity of vection that is a commonly measured variable in vection research^7,32^. To do so, after treatment, the participants would be asked to rate the intensity of vection on a given scale.

Another factor to pay attention to in future studies resides in the fact that in the Water condition two layers of transparent material sit between the screen of the phone and the eyes of the participants, namely the transparent plastic of the diving mask and a layer of water between the mask and the phone. These two layers are not present in the Ground condition and their impact should be investigated while tapping into research on vision through transparent layers^73^ and refractive structure^74^.

Scholars in the field of vection have shown how complex the relationship between vection, VIMS and presence is. While this study measured these three variables and looked into their relationships, nothing significant was found. In our future study, we will keep investigating how these three variables impact each other.

In this study, the visual and auditory inputs were the same across conditions while the proprioceptive cues differed between conditions. It would be valuable in future studies to independently examine the auditory and visual inputs in order to better understand how each of them contribute to vection, VIMS and presence.

## Conclusion

For almost 150 years, perception scholars have been creative in finding ways to investigate the role of different sensory inputs on vection. This paper opens new possibilities in how to study vection in a new environment. The findings discussed above present new research questions that may prove interesting to scholars in this field. The methodology reported here will be valuable in making the aquatic environment a new component of the vection research field. Developing, testing, and implementing these new tools raises practical challenges, and we believe this work will serve as a blueprint for future research.

Vection has been a central question in the field of VR and VIMS has been a constant struggle, with the goal of making vection more seamless in VR. Moreover, the field of VR has been extremely creative in developing immersive experiences. Aquatic VR presents a new frontier in the field of virtual immersion that can be explored thanks to the equipment used in this study. While more research is needed, aquatic VR might trigger vection without the associated VIMS and thus make VR activities comfortable enough to keep pushing the boundaries of what can be experienced in VR.

This first empirical work investigating the impact of a VR experience for participants floating in water found that vection is enhanced by the aquatic environment. This study also suggests that presence and VIMS are not affected by the aquatic environment. The encouraging results concerning vection in water call for further exploration of the underlying sensory mechanisms involved in this enhanced vection. The findings also challenge the use of well-established tools to measure VIMS in a novel situation. In sum, this study raises a wide range of new empirical and methodological questions concerning the perceptual experiences associated with water immersion. This constitutes an encouraging invitation to the fields of vection and VR to explore further the affordances of being immersed in water while simultaneously immersed in a virtual environment.

## Method

### Participants

The study aimed at collecting data from 30 participants in each condition. Flyers and emails recruiting participants comfortable with a snorkel were distributed in the community surrounding the pool where the study would take place. The study was planned to run from February until the end of March 2020 and the time slots filled in quickly. After collecting data from 39 participants, the pandemic hit and the study had to be stopped, leaving fewer data points than expected. One participant decided to discontinue their participation and was excluded from the analysis. The final sample (n = 38) consisted of 23 females (n = 12 in Ground condition, n = 11 in Water condition) and 15 males (n = 8 in Ground condition, n = 7 in Water condition). Their age ranged from 18 to 45 years old (*M* = 24.8, *SD* = 7.5). Twenty identified as White Caucasian (52.6%), 6 identified as Chinese (15.8%), 3 identified as multiracial (7.9%), 2 identified as African American (5.3%), 2 identified as Indian (5.3%), 2 identified as Japanese (5.3%), 1 identified as Latino (2.6%), 1 identified as Middle Eastern (2.6%) and 1 declined to answer (2.6%). All the participants volunteered for the experiment and were paid an honorarium for their participation. All protocols were carried out in accordance with current guidelines and regulations. The methods were approved by Stanford’s Institutional Review Board and we obtained informed consent from all the participants.

### Equipment

In both conditions, the virtual environment was displayed on a Samsung Galaxy S8 SM-G950F mobile phone which runs a custom operating system designed by Ballast Technologies, Inc. The operating system allows for seamless activation of the VR content experiences. The VR experience is activated and stopped by touching a near-field communication (NFC) card to the back of the phone (encased in different gears depending on the condition, see below) allowing for operation without the need for menu navigation or user interfaces. The researchers used two NFC cards, each assigned a special code to start or stop the VR experience entitled OceanDIVR, a 5-min long VR activity developed by Ballast Technologies, Inc. In this VR activity, the participant embarks on an approximately 285-m long drift dive (distance along the x-axis: 270.5 m, distance along the y-axis, − 90.5 m) in the ocean, visiting underwater wrecks, caves and submarines while encountering manta rays, sharks, and a pod of singing humpback whales. The virtual dive simulates a self-motion of 1.77 m per second.

#### Ground condition

In the Ground condition, the Samsung S8 mobile phone displaying the VR activity was encased in the Samsung Gear VR headset compatible with the Samsung 8. Headphones were connected to the phone for audio input.

#### Water condition

**Figure 3:**
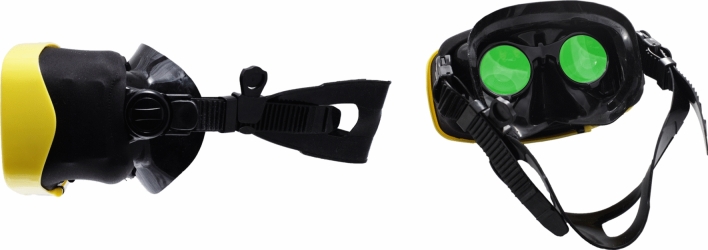
DIVR headset. Image courtesy of Ballast Technologies, Inc.

The Samsung S8 mobile phone displaying the VR activity, encased in a waterproof enclosure, was placed in the DIVR headset, designed to incorporate water between the lenses and the display to minimize buoyancy of the headset and maximize visual clarity while submerged in water (Fig. 3). The sound traveled from the phone to the participants through the water.

**Figure 4:**
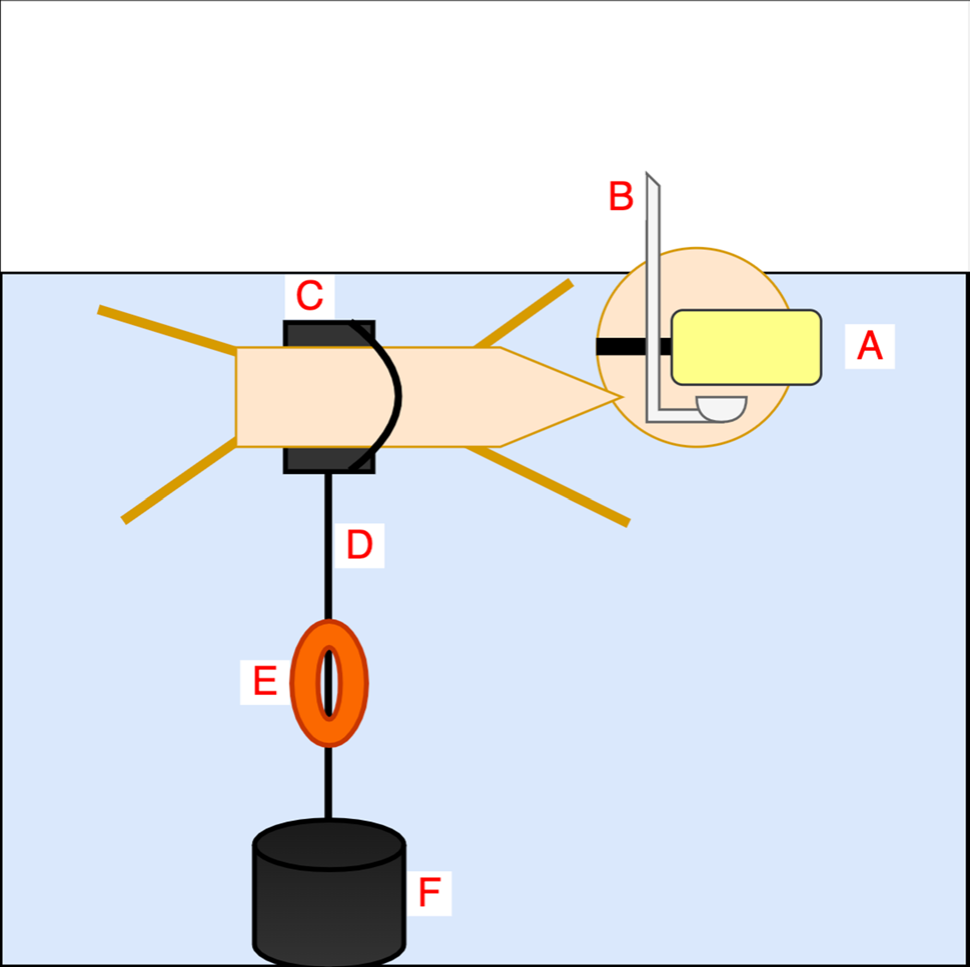
Equipment for the Water condition. The participant was equipped with the DIVR headset (**A**) encasing a Samsung S8 mobile phone displaying the VR activity. During the activity, the participant with their head underwater breathed through a snorkel (**B**). In order to keep the participant positively buoyant, a flotation belt (**C**) was placed around their hips. The flotation belt clipped into an elastic tether (**D**) attached to a 40-lb anchor placed on the pool floor (**F**). A pair of orange armbands (**E**) were attached to the tether mid-water and were used as an orange mark to draw the participant's attention to where they would start the VR activity before entering the water.

The participants were equipped with a flotation belt around their hips and a snorkel so they could comfortably float on their stomach. Each participant was assigned an interchangeable mouthpiece that was sanitized and recycled after use. In order to prevent the participants from swimming around the pool or drifting away, the flotation belt clipped into an elastic tether which was attached to an anchor placed on the pool floor. The elastic tether had a medium resistance tension, so that the users would not be aware of any abrupt tension on the tether. With their vision obscured by the headset, the participants were not aware of being held in place by the tether and anchor as the researcher hooked the tether to the flotation belt after the participant put the headset on. Figure 4 illustrates the participants with the equipment.

### Procedure

**Figure 5:**
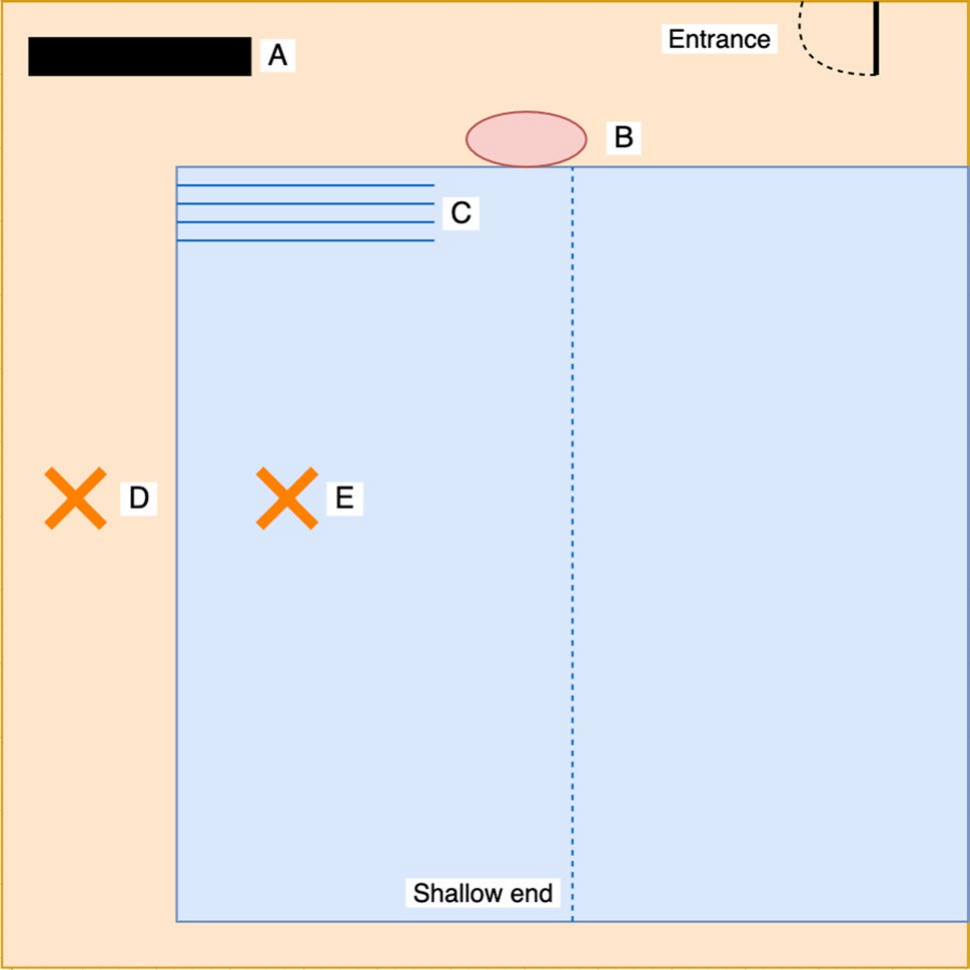
Bird’s eye view of the pool where the study was conducted. The participants reached the deck of the pool through the entrance and walked toward the bench (**A**) where they completed the pre-survey. Participants assigned to the Ground condition were invited to walk to the orange mark on the ground (**D**) where they started their VR experience. Participants assigned to the Water condition were first invited to go to the edge of the pool (**B**) where the researcher in charge of the Water condition briefed them and presented the equipment. The participants then entered the water through the stairs (**C**) and swam to the orange mark (**E**) where they started their VR experience.

The study was run in February 2020. Participants who signed up for the study were invited to meet the researcher on the deck of a local swimming pool they had free access to (Fig. 5). Upon their arrival, participants were instructed to read and sign the consent form along with a form for paid participation. Since international participants were expected to join the study, the researcher asked them which unit of length they were most comfortable with: meters or yards. By letting the participants choose, we tried to limit the inaccuracy due to a lack of familiarity with an imposed unit of length. Depending on the participants' choice, the researcher presented a ruler of 1 m or 1 yard and instructed the participants to look at it for 5 s. The participants were then asked to sit down and, wearing headphones to avoid background noise distraction, took a pre-survey including demographic questions. After completing the pre-survey, the participants were assigned to their condition; Ground or Water.

#### Ground condition

The researcher pointed at an orange duct tape cross on the floor situated approximately 15 m away and asked the participant: “Do you see the orange mark on the floor?” After receiving an affirmative answer from the participant, the researcher said: “This is where you will start your VR experience” and invited the participant to follow them to the orange mark. The participant was informed that they would watch a 5-min video with a VR headset. The researcher explained that the participant could look all around during the experience. The researcher also made clear that the participant could stop the activity at any point if anything was uncomfortable. Finally, the researcher instructed the participant to indicate when the VR activity was over but to not remove the headset on their own. The researcher helped the participant adjust the headset and placed the unplugged headphones on the participant head then placed the activation card labeled “Ocean DIVR” in front of the headset to start the experience. When the researcher heard the sound indicating that Ocean DIVR had started, they plugged the headphones into the headset. The researcher stayed next to the participant during the duration of the VR experience for safety purposes.

When the participant informed the researcher that the activity was over, the researcher removed the headphones and reminded the participant to keep the headset on. Then the researcher read the following instructions to the participant:

*Before removing the headset, I would like you to think about your position in the real world. Can you tell me which unit of length you selected earlier?*

The participant then reminded the researcher whether they selected meters or yards.

*Remember that I showed you the length of a [meter or yard]. How far away in the physical world do you think you are from the orange mark where you started the VR activity?*

The participant orally gave their answer that was recorded by the researcher. Then, the researcher removed the headset and invited the participant to walk back to the bench to take a second survey while wearing headphones to avoid background noise distraction. Finally, the participant was thanked for their participation and informed that they would receive their compensation by the end of the study.

#### Water condition

The participant was invited to come to the edge of the pool to be briefed about the experience. The researcher informed them that they would watch a 5-min video with a VR headset while floating horizontally in the pool. The researcher told the participants that they could look all around during the experience. The researcher made clear that the participant could stop the activity at any point if anything was uncomfortable. The headset, the snorkel, and the flotation belt were shown to the participant along with a picture of a fully-equipped individual in the water so that the participant could picture themselves in the situation. The participant was also told that the researcher would hook them up to a safety line that will help them follow the participant around and keep them safe. The participant was also informed that the procedure would include minimal physical contact such as holding their hand in order to help the participant maintain their balance when moving between the floating and standing position in water. The researcher asked if they would be okay with this minimal physical contact and all participants agreed. The participant was then given the opportunity to ask questions. Finally, the researcher invited the participants to get ready to go in the water by removing the clothes they were wearing on top of their swim suit.

Instead of an orange duct-tape cross in the Ground condition, the researcher pointed at two orange armbands hooked to the anchor and situated approximately 15 m away and asked the participant: “Do you see the orange mark in the water?” After receiving an affirmative answer from the participant, the researcher said: “This is where you will start your VR experience” and invited the participant to follow them to the orange armbands in the pool.

Before starting the VR activity, the researcher assisted the participant in putting the headset on and adjusting it. The participant was then instructed to place the snorkel in their mouth and to float horizontally with their head underwater to test the mask and the snorkel. If the equipment did not feel right, the researcher would assist in adjusting the seal of the mask, tightening the mask or making sure the snorkel was correctly positioned. Once the participant felt like the equipment was positioned correctly, the researcher started the Ocean DIVR experience by placing the activation card in front of the headset, and invited the participant to go back to the horizontal floating position. At that moment, the researcher hooked up the flotation belt to the anchor and stayed next to the participant during the duration of the VR experience for safety purposes. When the participant tried to stand up at the end of the activity, the researcher grabbed their hand and helped them land on their feet without touching the anchor.

When the participant was securely standing, the researcher read the same script as in the Ground condition in order for the participant to estimate how far away from their starting point they thought they were standing. Then, the headset was removed and the participant was invited to exit the pool and to use the provided towels and robes to dry out and get warm before taking a second survey, while wearing the headphones to avoid background noise distraction. Finally, the participant was thanked for their participation and informed that they would receive their compensation by the end of the study.

### Measure

#### Vection

We measured vection through a perceived body displacement technique known as egocentric distance estimation. This technique quantifies how far away from the initial position participants thought they were when they completed the VR experience. Similar to the reference point method used by previous researchers^28^, the participants started the VR activity at a reference point (a large orange “X” on the floor for the Ground condition and two orange armbands floating midwater for the Water condition). At the end of the VR activity, before removing their headset, the participants were asked to estimate in a familiar unit of length, how far away they were standing from their reference point^75^.

#### Visually induced motion sickness (VIMS)

The Simulator Sickness Questionnaire (SSQ^64^:) was used to measure VIMS. This questionnaire has been frequently used in the field of VR and vection. SSQ includes 16 items surveying the occurrence of motion sickness symptoms such as nausea, eyestrain, stomach awareness and general discomfort rated on a 4-point Likert scale ranging from “not at all” to “severe”. The participants filled out the SSQ right after the experimental treatment. Kennedy et al.^64^ suggested clustering the items into three factors: Nausea, Oculomotor and Disorientation. This method was not satisfactory as the reliability of two out of the three scales were low (Nausea: Cronbach’s alpha = 0.26 [*M* = 0.25, *SD* = 0.22], Oculomotor: Cronbach’s alpha = 0.57 [*M* = 0.28, *SD* = 0.27], Disorientation: Cronbach’s alpha = 0.72 [*M* = 0.23, *SD* = 0.3]). Instead of using the three factors from Kennedy et al. (2003), and since this is an exploratory study, we took two approaches. First, as the overall reliability for the 16 items together was good (Cronbach’s alpha = 0.73 [*M* = 0.23, *SD* = 0.22]) we looked at the SSQ score (method previously used^76^). Second, we analyzed each item separately (as previously done^77^).

#### Presence

The presence scale included six items on a 5-point Likert-scale from “Not at all” to “Very strongly” and adapted from Nowak and Biocca^78^. This scale included items such as “How much did it feel as if you visited another place?” and “How much was the virtual world like the real world?” Since the experiment took place outdoors, an item inquired the extent to which the weather conditions distracted the participants. This item was reversed coded. The reliability of the scale was good with a Cronbach’s alpha = 0.81 (*M* = 3.66, *SD* = 0.59).

### Approval for human experiments

All the participants volunteered for the experiment and were paid an honorarium for their participation. All protocols were carried out in accordance with current guidelines and regulations. The methods were approved by Stanford’s Institutional Review Board and we obtained informed consent from all the participants.
